# Brain Tumor Segmentation Network with Multi-View Ensemble Discrimination and Kernel-Sharing Dilated Convolution

**DOI:** 10.3390/brainsci13040650

**Published:** 2023-04-11

**Authors:** Xin Guan, Yushan Zhao, Charles Okanda Nyatega, Qiang Li

**Affiliations:** 1 School of Microelectronics, Tianjin University, Tianjin 300072, China; 2 School of Electrical and Information Engineering, Tianjin University, Tianjin 300072, China

**Keywords:** brain tumor segmentation, hierarchical multi-view convolution, ensemble discrimination, feature similarity, various sizes, deep learning

## Abstract

Accurate segmentation of brain tumors from magnetic resonance 3D images (MRI) is critical for clinical decisions and surgical planning. Radiologists usually separate and analyze brain tumors by combining images of axial, coronal, and sagittal views. However, traditional convolutional neural network (CNN) models tend to use information from only a single view or one by one. Moreover, the existing models adopt a multi-branch structure with different-size convolution kernels in parallel to adapt to various tumor sizes. However, the difference in the convolution kernels’ parameters cannot precisely characterize the feature similarity of tumor lesion regions with various sizes, connectivity, and convexity. To address the above problems, we propose a hierarchical multi-view convolution method that decouples the standard 3D convolution into axial, coronal, and sagittal views to provide complementary-view features. Then, every pixel is classified by ensembling the discriminant results from the three views. Moreover, we propose a multi-branch kernel-sharing mechanism with a dilated rate to obtain parameter-consistent convolution kernels with different receptive fields. We use the BraTS2018 and BraTS2020 datasets for comparison experiments. The average Dice coefficients of the proposed network on the BraTS2020 dataset can reach 78.16%, 89.52%, and 83.05% for the enhancing tumor (ET), whole tumor (WT), and tumor core (TC), respectively, while the number of parameters is only 0.5 M. Compared with the baseline network for brain tumor segmentation, the accuracy was improved by 1.74%, 0.5%, and 2.19%, respectively.

## 1. Introduction

The precise determination of the boundaries of brain tumor areas from MRI is an important basis for physicians to diagnose, treat, surgically evaluate, and follow up on tumors. However, brain tumors have various shapes and complex boundaries; therefore, manual segmentation is time-consuming and labor-intensive, and it is challenging to guarantee segmentation accuracy. Automatic segmentation of brain tumors by computer can greatly improve imaging physicians’ efficiency and segmentation accuracy, which has significant clinical practical value.

With the rise of artificial intelligence, deep learning techniques are being widely used in the fields of image, information system, and natural language processing [1,2]. Among these, CNN, as one of the representative algorithms of deep learning, performs well in image-related tasks and has greatly promoted the development of image segmentation, classification, detection, and other technologies since its initial proposal [3]. Subsequently, a large number of excellent network models have emerged, including ResNet and DenseNet, and these have enriched the applications of convolutional neural networks in various fields [4]. Since convolutional neural networks can automatically learn representative and complex features directly from a dataset to train a network model with strong robustness and learning ability without inputting manually designed features, they are widely used in brain tumor segmentation. Currently, experts and scholars have proposed various effective automated brain tumor segmentation methods in the context of continuous development and the improvement of medical imaging equipment [5,6,7,8].

Magnetic resonance images of brain tumors are available in axial, coronal, and sagittal views, and brain tumors present significantly different information in the different views. According to the division of the way to utilize the MRI view of brain tumors, the current deep learning segmentation methods are mainly single-view methods with axial or multi-view methods with a particular focus on this view. For brain tumor segmentation methods that use 2D slices in the axial view, although the axial view can contain partial information about the brain, the complete lesion area cannot be observed from this view [9]. Further information on the location and shape of the brain tumor needs to be determined by combining the coronal and sagittal views [10]. In order to combine information from multiple views, some researchers decomposed the standard 3D convolution kernel into the axial intra-slice and inter-slice convolution kernels, which perform convolution operations in the axial plane and the view perpendicular to the axial plane, respectively [11]. However, the receptive fields of the two convolutions are not the same, and only two orientations of the view are used. The extracted 3D spatial information still has limitations. To combine the three-view information, another way is to split the 3D dataset into axial, coronal, and sagittal 2D sliced images and extract the features in each view slice separately using different 2D CNNs, which makes full use of the spatial information and further improves the segmentation accuracy [12]. However, individual models can often only perform limited feature extraction for images under a single view during the training process. For a single model, the complete feature extraction of the contextual image information relies on integrating each network. This processing of each view modeled independently before fusion ignores the correlation between each view slice and increases the complexity of the model.

In addition to taking full advantage of the axial, coronal, and sagittal views of the brain tumor, it is also necessary to consider using different-size convolution kernels to adapt brain tumor lesions of various sizes to improve the segmentation accuracy further. Due to the random size of brain tumors in MRI, segmentation models need to be adapted to lesions of different sizes. A large receptive field enables consideration of a larger range of contexts and more semantic information, which is crucial for processing large-size images of brain tumor lesions. In contrast, a small receptive field better captures the local detail information, facilitating a finer delineation of boundaries and more accurate predictions, especially for small brain tumor lesions. However, brain tumor segmentation models that use standard convolution for extracting features use only a single-size kernel per convolutional layer. This results in small and fixed receptive fields, limiting the ability of the network to represent features with varying lesion sizes [13] adaptively. Dilated convolution allows us to set different dilation rates for the traditional standard convolution kernel and add zero-value pixel points between the individual pixel points of the convolution kernel. This varies the size of the kernel and thus flexibly expands the receptive field of the convolution kernel. Dilated convolution is used to have a larger receptive field without changing the feature map size, and there is no need to use pooling for downsampling. In contrast, a single dilated convolution has a specific receptive field. Convolution kernels with small dilation rates can learn detailed information well but cannot learn contextual features at a larger scale. Convolution kernels with large dilated rates can extract features with large receptive fields but lose more detailed information [14]. Using a single kernel for feature extraction reduces the ability of the network to generalize objects of different sizes.

Therefore, in order to have different receptive fields while taking into account the local details and global semantic information, the pyramid structure of the feature extraction part [15,16] adopts the parallel method of multiple dilated rate convolution kernels. In the ASPP structure [17], multiple dilated convolutional layers with different dilation rates are used in parallel to represent targets of arbitrary sizes, and their outputs are combined to integrate the information extracted from various receptive fields. To some extent, this improves the robustness of the model to image scale variations, but it cannot adapt to targets with high feature similarity and various sizes. In addition, this feature pyramid structure obtains different sizes of receptive fields by using parallel branches with independent kernels. It also causes the computational cost to increase with the number of parallel branches.

### 1.1. Motivation

After exploring conventional CNN brain tumor segmentation methods, we found that these often use information from only a single view. However, physicians often combine information from three views for brain tumor segmentation: axial, coronal, and sagittal. Moreover, brain tumors and their subregions have complex and irregular border structures. The standard convolution kernel cannot automatically adapt to various tumor sizes, connectivity, and boundary concavity and extract similar features simultaneously. To address these issues, we propose an end-to-end 3D brain tumor segmentation network based on hierarchical multi-view convolution and kernel-sharing dilated convolution (MVKS-Net), where 3D multi-view convolution is inspired by physicians’ segmentation process, and kernel-sharing dilated convolution characterizes the similar textures in the irregular realm of brain tumors.

### 1.2. Contributions

The contributions of this study are as follows:

We propose an axial–coronal–sagittal fusion convolution (ACSF), which decouples the standard 3D convolution into convolutions on three orthogonal views: axial, coronal, and sagittal. Combined with the extracted image features of the axial, coronal, and sagittal planes, the determination of the category of pixels can be further optimized by integrating two additional discriminations of the pixels in brain tumor images.

We propose a hierarchical decoupled multi-scale fusion module based on ACSF convolution. By incorporating short connections with residual-like structures between multi-view convolutional blocks for multi-scale feature fusion, the image information can be promoted to flow smoothly through each feature subgroup, and the receptive field of the module will gradually become larger, thus improving the perception of 3D spatial contextual information of the network.

We propose a kernel-sharing convolution with dilated rates (KSDC). Multiple branches with different dilation rates share a single kernel, which can simultaneously learn brain tumor features with different sizes and high feature similarity. This can better represent the complex boundaries and improve segmentation accuracy. In addition, kernel sharing significantly reduces computational costs.

The remainder of this study is structured as follows. Related works are discussed in Section 2. Section 3 describes the framework of brain tumor segmentation networks. Section 4 provides the experimental analysis results and compares them with current advanced methods. In Section 5, we summarize the proposed method and discuss its prospects. In Section 6, we present future research directions.

## 2. Related Work

The complete spatial context information of the 3D MRI image is essential for the accurate segmentation of brain tumor contours. Combining image information from multiple views helps to improve segmentation accuracy further. In addition, the receptive field of the convolution kernel represents the context range. A large receptive field enables the network to consider a broader context and more semantic information, and a smaller receptive field helps to capture the local details, which also helps generate finer brain tumor boundaries and more accurate predictions. Therefore, this section is mainly introduced from two parts: multi-view fusion and a model using different receptive fields to extract the features of various size targets.

### 2.1. Multi-View Fusion

In recent years, fully convolutional neural networks have been favored by researchers in medical image segmentation, among which the U-shaped architecture has good performance in brain tumor image segmentation. However, these methods are still dominated by a single view. In order to improve the ability to capture convolutional network spatial information, some research works adopt the idea of multi-view fusion for brain tumor image segmentation. Ding et al. [9] propose a multi-view dynamic fusion framework, which slices normalized 3D image data from the axial, coronal, and sagittal views into 2D images and introduces a fusion multi-view loss to promote the training process of multi-view learning networks during network training. This method dynamically fuses brain tumor images from different views. It achieves good results in fine segmentation of whole tumors. Still, the standard two-dimensional convolution cannot fully use the 3D information from the MRI data, and the network structure is relatively complex.

The multi-view idea has also been applied to cascading convolutional neural network structures, using three networks to stratify the whole tumor, tumor core, enhanced tumor, in turn. The three networks were trained on the three orthogonal views, and the average of the prediction results of each network from the three vertical views was taken as the final result, achieving a competitive performance. However, this fusion operation ignores the importance of the information contained in each view, and the cascading structure requires longer training and testing times [11,18,19]. Pan et al. [20] processed the scanned axial, coronal, and sagittal views using a separable convolution strategy. The architecture of each view is designed in a multi-scale manner, from coarse to fine, to capture subtle differences and gain a diversity of receptive fields. Compared with 2D and 3D networks, this network can retain certain spatial information and reduce training parameters, respectively, but the extracted 3D information still has limitations. Some experts and scholars adopt a multi-view fusion strategy that integrates multiple 2D CNNs [21,22], and the spatial context information of 3D MR images is essential for brain tumor segmentation. Still, this integration of numerous 2D CNNs cannot fully use the 3D spatial information of brain tumor images.

Although these methods have achieved good results, there are still certain limitations. First, multiple networks are integrated for multi-view fusion, which is complex and inefficient. In addition, the use of 2D networks ignores the continuity between slices and cannot fully use MRI data information. Segmentation operations along the sagittal, coronal, and axial directions obtain three trained models, respectively, which ignore the multi-view results’ appearance, and the spatial consistency paper adopts a 3D convolutional neural network and applies the multi-view idea to the convolutional block [23]. Instead of explicitly treating the input 3D image as three orthogonal two-dimensional flat images, we directly split the 3 × 3 × 3 convolution kernel into three parts of sizes 3 × 3 × 1, 3 × 1 × 3, and 1 × 3 × 3, which are operated on the convolution kernel, and then acted on the three orthogonal views of the axial, coronal, and sagittal planes of the brain tumor to obtain a view-based 3D representation of each three-dimensional image. The additional two discriminations of the brain tumor image pixels further optimize the judgment of the category of pixels, and it improves the ability of the model to capture multi-view information from images.

### 2.2. Multi-Scale Receptive-Field Feature Extraction Model

Due to the different sizes of brain tumors in MRI images, adequate characterization of tumors at different scales is critical. This requires network models that can process small-size brain tumor details and large-size brain tumor information to better process brain tumor images of any size.

In order to achieve scale-adaptive characterization of tumors, existing studies have started by using parallel structures of multiple standard convolution kernels of different sizes or by introducing dilated convolution [24]. Some researchers used different-size parallel structures of standard convolution to adapt to various brain tumors for feature extraction, such as the Inception structure. Zhang et al. [13] used multi-scale feature extraction blocks instead of standard convolution in encoders to extract and aggregate valid information from different receptive fields, the network maximizes the aggregation of multi-level features at different scales to achieve complementary advantages between features, but standard two-dimensional convolution cannot make full use of the context information of the spatial dimensions in 3D images. Punn et al. [25] divided the brain tumor segmentation task into multimodal fusion, tumor extraction, and tumor segmentation, leveraging the advantages of Inception convolution and the 3D U-Net architecture to improve the understanding of deep patterns related to tumor regions. Hussain et al. [26] used kernels of different sizes from 5 × 5 to 13 × 13 to form three parallel paths, using larger kernels to obtain more contextual information and smaller kernels to model the correlation between pixels, yielding excellent results in tumor cores and enhancing tumor regions. However, there is still room for improvement in the feature extraction of whole tumors. Khened et al. [27] combined the parallel structure of the Inception model to increase the receptive field by removing the maximum pooled branch and introducing a larger convolution kernel and obtained high segmentation accuracy, but the large convolution kernel was inadequate for the extraction of more fine brain tumor details. This parallel structure of multiple standard convolutions, in which each branch uses standard convolutions of different sizes, reduces the image resolution when extracting the product layer by layer, resulting in the loss of some key and tiny features of the tumor’s internal tissue during propagation.

Compared with standard convolution, dilated convolution adds zero-value pixels between the pixels of the convolution kernel, which can have a larger receptive field without changing the image size. Therefore, some researchers use convolution kernel or pooling operations with different dilated rates and multiple receptive fields to detect input features, accommodate brain tumors of various sizes, and encode multi-scale contextual information [28]. The lightweight 3D-ESPNet [29] extends ESPNet [30] to the 3D brain tumor segmentation task for the first time, which is based on the “reduce-split-transform-merge” decomposition idea, through the efficient spatial pyramid ESP module for feature extraction, and finally, introduces four parallel convolution kernels of different sizes in the pyramid refinement module. This method obtains multi-scale receptive fields without increasing the number of parameters, but the “reduced” and “split” feature maps will reduce the segmentation performance of the network. On this basis, some researchers have improved this parallel branch structure with multiple dilated convolutions in position or in combination with other structures. Among these, Ahmad et al. [17] use a residual-dilated module in the coding layer, in which convolution kernels with different dilated rates are connected in a series to increase the receptive field and add residual connections to extract image features. Additionally, dense ASPP modules are combined to save more contextual information about small-size tumors on each level of the encoder path. However, the receptive field is still limited, and it is impossible to simultaneously extract tumors of different scales with similar characteristics. To further extract multi-scale image features, DFP-ResUNet [15] uses a spatial-expansion feature-pyramid module composed of three parallel dilated convolutional layers at the bottom of the U-shaped network and sets the dilated rates to 4, 8, and 12. It improves the extraction ability of the image features of tumors of different sizes and better extracts the features of the different positions of the image by expanding the receptive field of the convolutional layer, but the performance of the proposed method in the whole tumor region needs to be improved.

In order to enhance the ability of the whole model to distinguish tumors of different sizes, AFPNet [16] uses single-step 3D dilated convolution instead of pooling and stride and builds a backbone network for feature learning, which solves the problem of spatial information loss caused by repeated pooling and stride. It designs a 3D dilated convolution integral-layer feature pyramid and adds it to the end of the backbone network, which further improves the segmentation accuracy of enhanced tumor and tumor core by combining with the contextual features, but it cannot extract brain tumors with very complex boundaries well. RDAU-Net [31] adds an extended feature pyramid module with an attention mechanism between the encoder and decoder, effectively obtaining feature maps of various sizes through different dilated convolutions while extracting useful information about channels and spaces. It solves the problem of traditional U-Net networks being unable to extract the multi-scale features of images. Still, this method does not have high segmentation accuracy in the whole tumor region. DMF-Net [32] is based on multi-fiber units, using efficient group convolution and 3D dilated convolution to establish multi-scale feature representation. The proposed structure can maintain high-precision brain tumor segmentation while greatly reducing the computational cost. Still, it cannot fully extract the brain tumor features with high similarity in various sizes in an image. The parallel strategy of multiple dilated convolution can adapt to brain tumor lesions of different sizes; however, the brain tumors in MR images have different sizes and very complex boundaries. When the similarity of the feature representations is high, the parallel dilated convolution cannot sufficiently represent these features with high similarity, which limits the improvement of brain tumor segmentation accuracy.

To solve the above problems, we apply the kernel-sharing dilated convolution (KSDC) to the brain tumor segmentation task, in which multiple branches with different dilated rates can effectively share a single kernel. Through the sharing mechanism, the convolution kernels of various receptive fields can be obtained at the same time, and the weight parameters learned by different receptive field branches are consistent, which can adapt to brain tumor features of different sizes and similar characteristics. It improves the representation of shared kernels, thereby improving the segmentation accuracy of brain tumors.

## 3. Method

**Figure 1:**
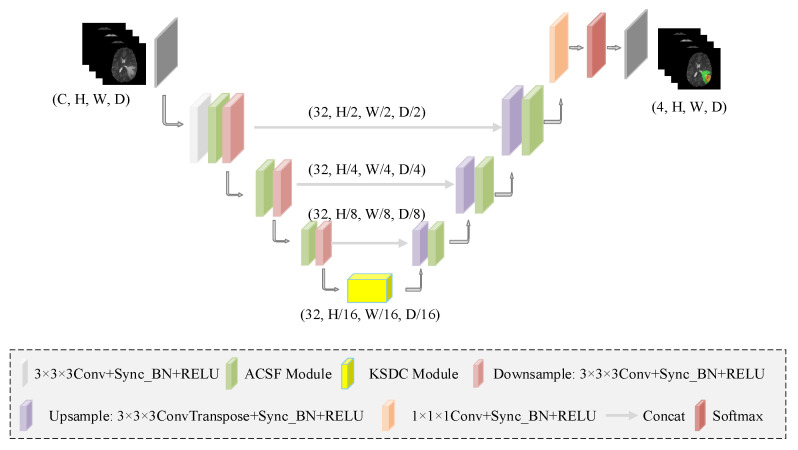
The complete network structure of MVKS-Net. C denotes the channel number of feature maps, H denotes the height of feature maps, W denotes the width of feature maps, and D denotes the depth of feature maps, respectively. We design a network with an encoding–decoding structure, which contains a hierarchical multi-view module based on axial–coronal–sagittal fusion (ACSF) convolution to provide complementary view features and kernel-sharing dilated convolution (KSDC) to obtain parameter-consistent convolution kernels with different receptive fields.

The proposed 3D brain tumor segmentation network architecture of multi-view fusion convolution and kernel-sharing convolution (MVKS-Net) is shown in Figure 1. The main body of the network consists of hierarchical multi-view fusion convolution modules and kernel-sharing dilated convolution modules. Each layer is set up with 32 constant channels. The input to the network is a block of 3D images after four modalities of brain tumors are stitched. Each image block has a size of 128 × 128 × 128.

In the feature encoding stage, the input image uses 3 × 3 × 3 convolution, and the 4-channel image block is processed as a 32-channel image block with a size of 64 × 64 × 64. Then, the ACSF convolution module adaptively performs feature extraction under the axial plane, coronal plane, and sagittal plane of the 3D image block, further improving the model’s ability to capture the multi-view spatial information of the image. At the same time, after each convolution operation, synchronized batch normalization and ReLU function processing are performed. The downsampling uses 3 × 3 × 3 convolution with stride 2. In the final stage of the encoder, through the KSDC module, different-size receptive fields are generated, and the input features are scanned multiple times to better extract the high-level semantic information of the image and adapt to brain tumor lesions of different sizes. The decoding stage uses trilinear interpolation to upsample the feature map. A skip connection is used between the encoder and decoder to concatenate the upsampled features with the encoder’s high-resolution features. The details of the network structure are shown in Table 1.

### 3.1. ACSF Convolution

**Figure 2:**
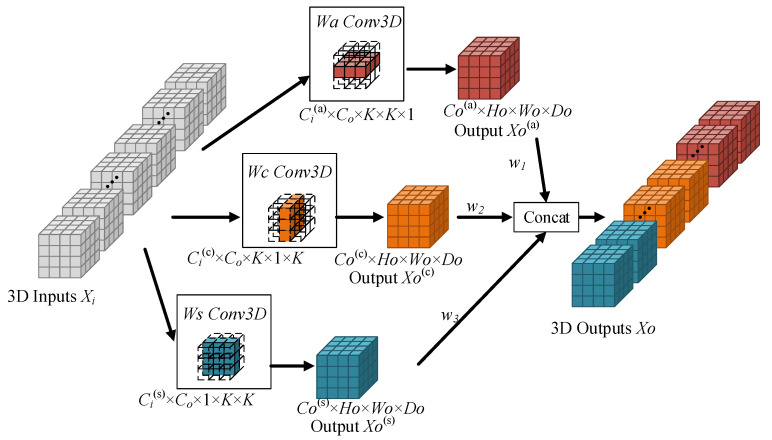
ACSF convolution.

In order to directly extract 3D spatial information from the axial, coronal, and sagittal views of MR images, we propose a new convolution method, namely axial–coronal–sagittal fusion (ACSF) convolution. ACSF convolution solves the 3D convolution integral into asymmetric convolution in the axial, coronal, and sagittal directions, and the specific implementation is shown in Figure 2. Suppose the 3D input image is $X_{i}\in R{}^{C_{i}\times H_{i}\times W_{i}\times D_{i}}$; the 3D output image is $X_{o}\in R{}^{C_{o}\times H_{o}\times W_{o}\times D_{o}}$, where $C_{i}$ and $C_{o}$ represent the input and output channels; and $H_{i}$, $W_{i}$, and $D_{i}$ represent the height, width, and depth of the input image. $H_{o}$, $W_{o}$, and $D_{o}$ represent the height, width, and depth of the output image, respectively. Instead of presenting the 3D image as a 2D image slice with a three-view plane, we split and reshaped the convolution kernel of 3 × 3 × 3 into three parts. We insert an extra dimension of size one at different indices, generating kernels of 3 × 3 × 1, 3 × 1 × 3, and 1 × 3 × 3.

By learning the characteristics of the three views of brain tumors, H-W, H-D, and W-D, respectively, the representation of each convolution based on a single view is obtained: $W_{a}\in R{}^{C_{i}^{\left(a\right)}\times C_{o}\times 3\times 3\times 1}$, $W_{c}\in R{}^{C_{i}^{\left(c\right)}\times C_{o}\times 3\times 1\times 3}$, and $W_{s}\in R{}^{C_{i}^{\left(s\right)}\times C_{o}\times 1\times 3\times 3}$, where $C_{i}^{\left(a\right)}+C_{i}^{\left(c\right)}+C_{i}^{\left(s\right)}=C_{i}$. With the adaptive weights ω1, ω2, and ω3 assigned to each branch, the three-dimensional features of the axial, coronal, and sagittal views are calculated:

(1) $$X_{o}^{\left(a\right)}=\omega 1\times \mathrm{Conv}3D\left(X_{i},W_{a}\right),$$

(2) $$X_{o}^{\left(c\right)}=\omega 2\times Conv3D\left(X_{i},W_{c}\right),$$

(3) $$X_{o}^{\left(s\right)}=\omega 3\times \mathrm{Conv}3D\left(X_{i},W_{s}\right),$$

where Conv3D is a function that stands for three-dimensional convolution operations. ω1, ω2, and ω3 give weight to each output view. This weighting strategy helps to automatically select the most valuable information from the different views and suppresses features that are not conducive to improving segmentation accuracy. Then, the three result feature maps are fused to form the output feature map. With the help of ACSF convolution, the integrated coronal and sagittal image features will discriminate the pixel points twice more, which will give further basis for the classification of the pixel points, improve the classification accuracy of the pixel points, and help the final accuracy of the model segmentation.

In addition, for a convolution kernel of size k, the parameters of ACSF convolution are about $3k^{2}$, while the parameters of standard 3D convolution are about $k^{3}$. Multi-view fusion convolution and standard 3D convolution have almost the same parameters when the convolution kernel size is 3, but when the convolution kernel size is greater than 3, the ACSF convolution will have a smaller number of parameters than the standard 3D convolution. This characteristic makes it possible to use large kernels.

### 3.2. Hierarchical Multi-View Fusion Module Based on ACSF Convolution

**Figure 3:**
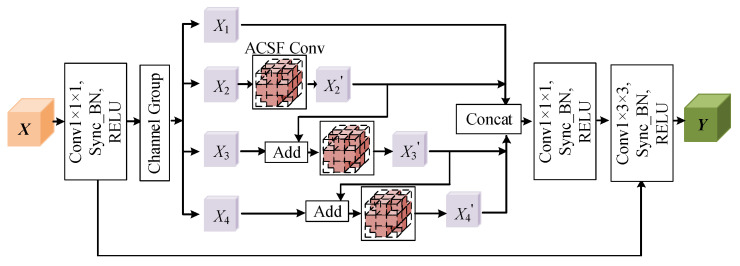
Hierarchical multi-view module based on ACSF convolution.

As shown in Figure 3, the input image *X* first performs a 1 × 1 × 1 convolution operation and then divides the 32 channels equally into four groups, corresponding to $X_{1}$, $X_{2}$, $X_{3}$, and $X_{4}$, each with eight channels. For the problem of insufficient feature extraction capability for each group, we add ACSF convolution units in parallel on different subgroups of the feature channel and perform ACSF convolution processing on subgroups $X_{2}$, $X_{3}$, and $X_{4}$. Finally, a short connection is applied between the corresponding subgroups.

(4) $$X_{2}^{\prime }=F_{ACSF}\left(X_{2}\right),$$

(5) $$X_{3}^{\prime }=F_{ACSF}\left(X_{3},X_{2}^{\prime }\right),$$

(6) $$X_{4}^{\prime }=F_{ACSF}\left(X_{4},X_{3}^{\prime }\right),$$

where $F_{ACSF}$ stands for ACSF convolution. The feature maps of the previous subgroup after the convolution operation will be accumulated as input to the next subgroup, which not only promotes the image information to flow smoothly through each feature subgroup, but, also, the module’s receptive field will keep increasing through the short connections between the subgroups, making it possible to capture richer multi-scale information of MR images from different views. We connect features $X_{1}$, $X_{2}^{\prime }$, $X_{3}^{\prime }$, and $X_{4}^{\prime }$, and, finally, we use a 1 × 1 × 1 convolution kernel to further adjust the feature maps and feature channels under the different receptive fields obtained by different groups. The residual connection between the input and output further improves the stability of the model information flow. Each layer of the network adopts the ACSF convolution module with a short connection for feature extraction, which further improves the segmentation accuracy of brain tumors.

### 3.3. Kernel-Sharing Dilated Convolution

**Figure 4:**
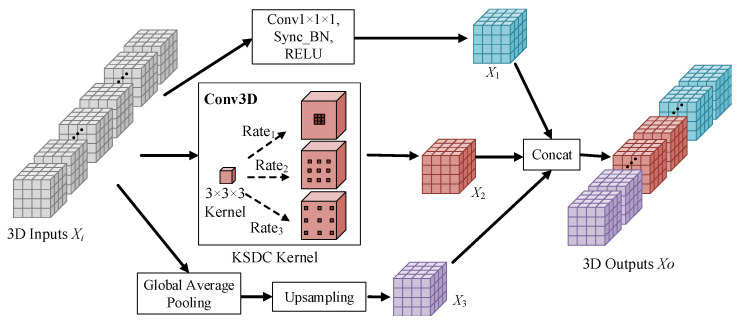
KSDC module.

When the features in the region of the tumor lesion and the boundaries in the MR image have high similarity, a multi-branch structure with multiple convolution kernels of various sizes in parallel cannot perform feature extraction well due to the inconsistent weight parameters learned by the large- and small-size convolution kernels. To address this problem, we propose a new mechanism, called kernel-sharing dilated convolution (KSDC). The overall structure of the proposed KSDC module is shown in Figure 4.

Suppose the input 3D image is $X_{i}\in R{}^{C_{i}\times H_{i}\times W_{i}\times D_{i}}$, where $C_{i}$ represents the input channel, $H_{i}$ represents the height of the input feature map, $W_{i}$ represents the width of the input feature map, and $D_{i}$ represents the depth of the input feature map.

The input image is processed in three parallel branches. The first branch performs 1 × 1 × 1 convolution to obtain the feature map $X_{1}$; the second branch performs pyramidal dilation convolution to obtain the feature map $X_{2}$, where the dilation rate is variable to generate different-size receptive fields; the third branch performs a global average pooling of the input image and an upsampling to recover the size of the feature map to obtain the feature map $X_{3}$. Finally, the three parts of the feature map are fused to obtain the output feature *Y*.

(7) $$X_{1}=F\left(X_{i},K_{1\times 1\times 1}\right),$$

(8) $$X_{2}=F\left(X_{i},K_{3\times 3\times 3},R\right),$$

(9) $$X_{3}=Up\left(G_{\mathrm{avg}}\left(X_{i}\right)\right),$$

where $K_{1\times 1\times 1}$ represents the convolution kernel of 1 × 1 × 1, $K_{3\times 3\times 3}$ represents the convolution kernel of 3 × 3 × 3, *R* represents the variable dilated rate, $G_{\mathrm{avg}\;}$ represents global average pooling, Up represents upsampling, $C_{o}$ represents the output channel, $H_{o}$ represents the height of the output feature map, $W_{o}$ represents the width of the output feature map, and $D_{o}$ represents the depth of the output feature map.

Multiple branches with different dilated rates share a kernel. The input feature maps are scanned multiple times by generating receptive fields of different sizes to adapt to lesion features of various sizes. Compared with the method of parallel multiple convolution kernels of different sizes, the sharing strategy proposed by us reduces the number of model parameters due to the sharing of convolution kernel parameters, which helps to reduce the computational cost. Sharing information increases the number of effective training samples, which improves the kernel’s representation ability and helps improve the segmentation performance.

## 4. Results

### 4.1. Datasets and Evaluation Indicators

**Figure 5:**
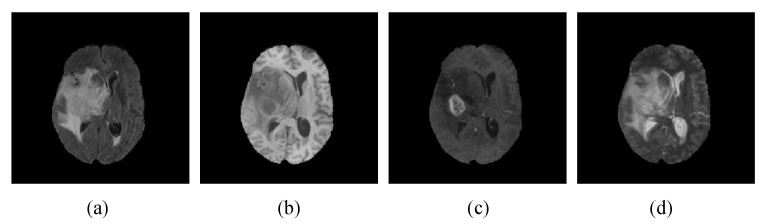
MRI images in different modalities: (**a**) Flair, (**b**) T1, (**c**) T1ce, and (**d**) T2.

We use the datasets from BraTS2018 and BraTS2020 [33,34] to verify the proposed method. Brain tumor MR images have three tumor tissue classes and one non-tumor category. The three tumor classes were enhanced tumors with a label value of four, peritumor edema with a label value of two, and necrotic and non-enhancing tumors with a label value of one. The BraTS2018 dataset contains 285 cases in the training datasets and 66 cases in the validation datasets. The BraTS2020 dataset contains 369 cases in the training datasets and 125 cases in the validation datasets. Each case includes four sequences of 3D MR data, corresponding to the four modalities of Flair, T1, Tlce, and T2, and each sequence contains 155 slides of size 240 × 240. The four modalities are shown in Figure 5. The three evaluation areas were whole tumor (corresponding to labels 1 + 2 + 4), tumor core (corresponding to labels 1 + 4), and enhanced tumor (corresponding to label 1). The results of brain tumor segmentation can be evaluated on the image processing portal of CBICA, an online evaluation platform. All cases were skull dissected and resampled to isotropic 1 mm^2^ resolution, while all four sequences of the same case were registered to the same anatomical template for calibration.

We take the segmentation accuracy and network complexity as the joint evaluation indices to comprehensively evaluate the brain tumor segmentation algorithm. The segmentation accuracy adopts two indicators: the Dice similarity coefficient (Dice) and the Hausdorff95 distance (HD). Dice indicates the similarity between the predicted and real tumors. The higher the value, the better the effect of the algorithm on tumor image segmentation. The expression is:

(10) $$Dice=2\times \frac{\left|P\cap T\right|}{\left|P|+|T\right|}.$$

The Hausdorff95 distance represents the maximum degree of mismatch between two sets of points. It is often used to measure the distance between standard segmentation and the predicted segmentation result from the segmentation algorithm. The expression is:

(11) $$\mathrm{HD}=d\left(P,T\right)=\max\left\{\underset{p\in P}{\sup}\underset{t\in T}{\inf}d\left(p,t\right),\underset{t\in T}{\sup}\underset{p\in P}{\inf}d\left(t,p\right)\right\},$$

where *P* and *T*, respectively, represent the voxel set of the tumor area and the real labeled area predicted by the network; *p* and *t*, respectively, represent the voxel point in the two voxel sets; and $d\left(p,t\right)$ is the distance between the two voxel points. Addtionally, inf represents the infimum of the set, and sup represents the supremum of the set. The Hausdorff95 distance is the maximum distance between the segmentation result and the true value. The smaller the absolute value, the more consistent the segmentation effect.

The computational complexity of the network model is quantitatively analyzed by the number of model parameters and the number of floating-point number operations. The parameter calculation formula is:

(12) $$\mathrm{Params}=k_{d}\times k_{h}\times k_{w}\times C_{in}\times C_{out}.$$

FLOPs are used to measure the time complexity of a network and are calculated as follows:

(13) $$\mathrm{FLOPs}=2\times \left(k_{d}\times k_{h}\times k_{w}\times C_{in}\right)\times C_{out}\times d\times h\times w,$$

where $k_{d}$, $k_{h}$, and $k_{w}$ represent the depth, height, and width of the convolution kernel $C_{in}$; $C_{out}$ indicates the number of input and output channels; and *d*, *h*, and *w* represent the depth, height, and width of the image, respectively.

### 4.2. Implementation Details

The experimental environment in this paper is as follows: CPU Intel® Core i9-9900X 3.5 GHZ, GPU GTX2080Ti (11 GB) × 4. We set the training to 900 epochs and the batch size to eight. We use the data augmentation of random cropping, random rotation, and random intensity offset at the same time to increase the number of training data and enhance the robustness and generalization ability of the deep learning training algorithms. To train the network model optimally, we employ the Adam optimizer with an initial learning rate of $10^{-3}$ and a weight decay of $10^{-5}$. Table 2 describes the parameters during model training.

Aiming at the problem that the gray value range of multimodal MR images is too different, which makes it difficult for the network to be quickly and effectively optimized, we preprocessed the data to normalize the multimodal MR images based on the mean and standard deviation of the same modal data from the original data. The processed data conform to the standard normal distribution, i.e., the mean is 0, the standard deviation is 1, and the transformation function is shown as follows:

(14) X=(x−u)/δ,

where *X* is the processed image, *x* is the initial image gray value, *u* is the gray mean of the same modal data for all cases, and δ is the standard deviation of the same modal data for all cases. The 3D images used during model training are randomly cropped to a size of 128 × 128 × 128 as input.

To solve the problem of data imbalance, our method adopts the generalized dice loss (*GDL*) [35] function, which alleviates the disadvantage of Dice loss [36] for detecting small targets by introducing weights.

(15) $$GDL=1-2\times \frac{\sum_{l=1}^{L}w_{l}\sum_{n=1}^{N}p_{ln}t_{ln}}{\sum_{l=1}^{L}w_{l}\sum_{n=1}^{N}\left(p_{ln}+t_{ln}\right)},$$

(16) $$w_{l}=\frac{1}{{\left(\sum_{n=1}^{N}t_{ln}\right)}^{2}},$$

where $w_{l}$ represents the weight of each category, $p_{\mathrm{In}\;}$ represents the value of voxel *n* in the predicted category *l*, and $t_{ln}$ represents the corresponding ground truth value. *L* and *N* represent the total number of categories and the total number of voxels, respectively.

### 4.3. Experimental Results and Analysis

#### 4.3.1. Comparison of HDC-Net and MVKS-Net Boxplots

**Figure 6:**
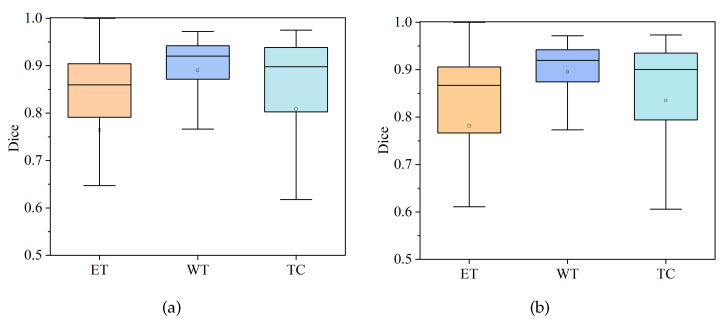
Dice coefficient boxplot on the BraTS2020 validation set. (**a**) stands for HDC-Net, and (**b**) stands for MVKS-Net.

**Figure 7:**
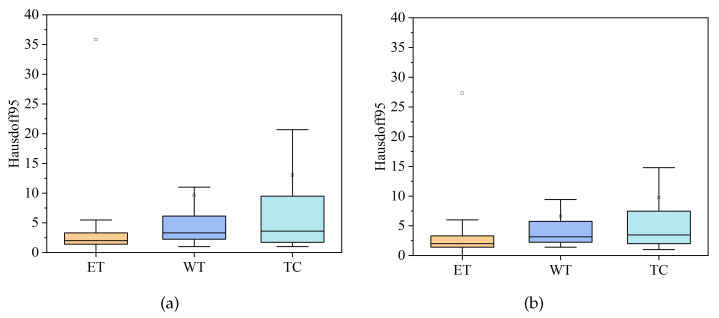
The boxplot of Hausdorff95 distance on the BraTS2020 validation set. (**a**) stands for HDC-Net, and (**b**) stands for MVKS-Net.

In order to show the distribution characteristics of HDC-Net [37] and the MVKS-Net segmentation results, this paper compares the segmentation results of ET, WT, and TC with a boxplot. Boxplots of the Dice coefficient and the Hausdorff95 distance for all cases in the validation set in the three tumor regions are shown in Figure 6 and Figure 7. The top and bottom short lines represent the maximum and minimum values of the data. The top and bottom of the box represent the upper and lower quartiles. The lines in the box represent the median of the data, and the points represent the average of the data. As can be seen from Figure 6 and Figure 7, the average Dice coefficient of MVKS-Net is higher than that of HDC-Net, which means that the proposed network shows better segmentation performance. For the Hausdorff95 distance, the MVKS-Net results are distributed in the ET and WT regions, and especially in the TC regions, and the discreteness of the MVKS-Net results is significantly better than those of HDC-Net. Overall, MVKS-Net has better segmentation accuracy and less discreteness than HDC-Net.

#### 4.3.2. Effect of Initial Learning Rate on Segmentation Performance

**Figure 8:**
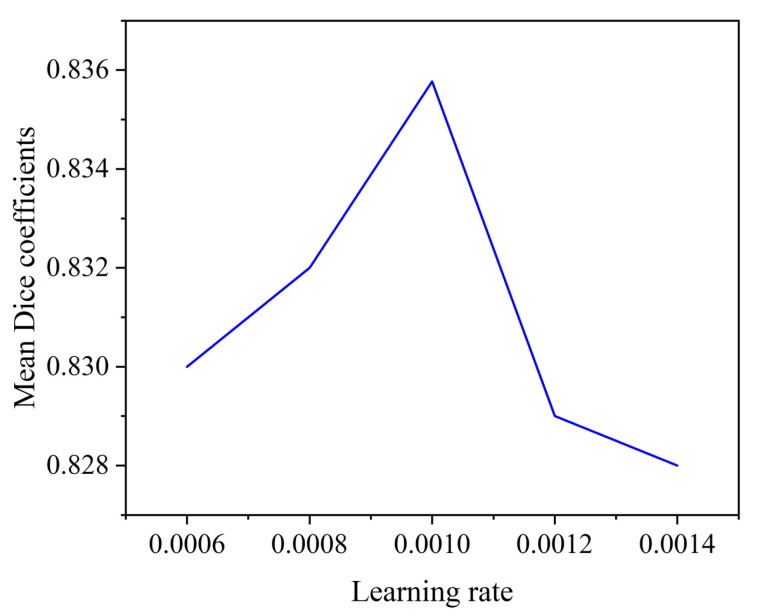
Average segmentation Dice coefficients of MVKS-Net at different learning rates.

The initial learning rate is a critical hyperparameter in model training. If it is set too small, it will converge slowly; if the setting is too large, the loss will fluctuate or even become larger, which will affect the final segmentation result of our segmentation network. Therefore, we design experiments to explore the effect of the initial learning rate on model performance, and the results are shown in Figure 8. Specifically, we set the initial learning rates of $0.6\times 10^{-3}$, $0.8\times 10^{-3}$, $1\times 10^{-3}$, $1.2\times 10^{-3}$, and $1.4\times 10^{-3}$ for our experiments, and we use the average Dice coefficient of the three regions as the evaluation index.

As shown in Figure 8, as the initial learning rate increases from $0.6\times 10^{-3}$ to $1\times 10^{-3}$, the average Dice coefficient also increases. When the learning rate is $1\times 10^{-3}$, the model achieves the best segmentation result. However, when the learning rate is greater than $1\times 10^{-3}$, the average Dice coefficient changes more drastically and decreases as the initial learning rate increases. Therefore, we choose an initial learning rate of $1\times 10^{-3}$. Then during the training process, the learning rate will be adjusted adaptively to converge to the optimal segmentation effect as soon as possible.

#### 4.3.3. Ablation Experimental Analysis

All ablation experiments were performed on the BraTS2020 validation set, and the Dice coefficient, Hausdorff95 distance, number of parameters, and FLOPs evaluation metrics of the experimental results are shown in Table 3, where ET indicates the enhanced tumor area, WT indicates the whole tumor area, and TC indicates the tumor core area. This paper uses the HDC network as the baseline model and adds different improvement strategies to observe the segmentation effect.

To verify the effectiveness of the ACSF module, we compare the performance of HDC-Net and HDC + ACSF. The use of the ACSF module increases the number of parameters and the computation of the model slightly, but the segmentation performance improves significantly, with Dice_ET improving by 1.31%, Dice_WT improving by 0.44%, and Dice_TC improving by 1.34%. This is because the HDC module decomposes two 3 × 3 × 3 convolutions into three 3 × 3 × 1 convolutions and one 1 × 3 × 3 convolution, which can focus the feature extraction into only one main view under a single network, while our method can extract features in three axial–coronal–sagittal views, which helps with accurate segmentation of brain tumors.

On the other hand, the use of the KSDC module improves Dice_ET by 1.11%, Dice_WT by 0.09%, and Dice_TC by 0.48%, which indicates that the proposed KSDC can improve segmentation performance to a great extent. With the simultaneous addition of ACSF and KSDC, the best segmentation results were obtained for the Dice coefficients and Hausdorff95 values in the ET, WT, and TC regions, where the Dice coefficients improved by 1.74%, 0.5%, and 2.19%, respectively, and the Hausdorff95 values decreased by 11.24 mm, 1.99 mm, and 2.98 mm, respectively.

**Figure 9:**
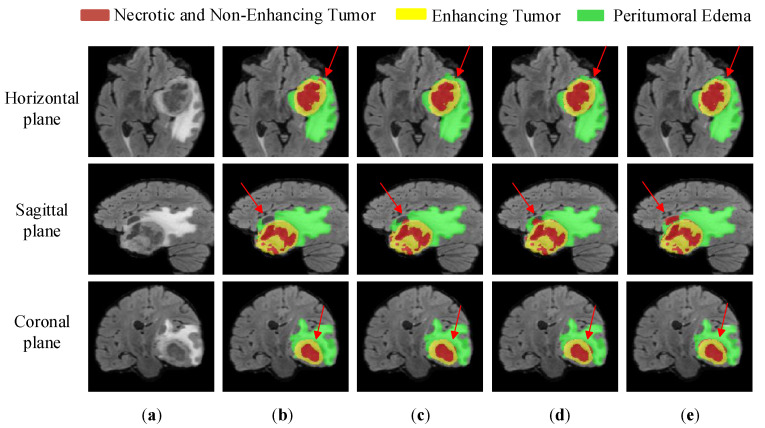
Segmentation results of the ablation experiment. (**a**) is the modality of Flair, (**b**) stands for HDC-Net, (**c**) stands for HDC + KSDC, (**d**) stands for HDC + KSDC + ACSF, (**e**) stands for ground truth. The yellow area is the enhancing tumor area. The red area is the necrotic and non-enhancing area. The green area is the peritumoral edema area.

Figure 9 is an example image of the segmentation results of the ablation experiment in three views, in which the red color indicates the necrotic area and the non-enhanced tumor nucleus area, the green color indicates the edematous area, and the yellow color indicates the enhanced tumor area. The example diagram uses the BraTS20_156 case, and the 3D coordinates are taken as 65, 155, and 128. As shown in the figure, compared with the true-value map, the second column HDC-Net segmentation map has the problems of label classification error and different sizes of sub-regions and ground truth regions, where the label classification error problem exists in the horizontal, sagittal, and coronal planes. After adding the KSDC module, the tag classification error problem is greatly reduced, but there are still inconsistencies between the sub-region size and the true-value region size. With the addition of both the ACSF module and the KSDC module, our network further improves the ability to identify the boundaries of tumor subregions, especially the edge details of necrotic areas. These segmentation figures show that the MVKS-Net segmentation maps designed in this paper are closer to the ground truth and also show the effectiveness of the KSDC module and the ACSF module proposed in this paper.

#### 4.3.4. Comparative Experiments and Analysis

To fully verify the effectiveness of MVKS-Net, comparative experiments were carried out on the BraTS2018 and BraTS2020 datasets with other high-performance models. Using the validation dataset of BraTS2020, MVKS-Net is compared with other advanced lightweight and non-lightweight networks. The segmentation results of all network models are shown in Table 4 and Table 5.

It can be seen from Table 4 that compared with the advanced lightweight network DMF-Net, the Dice coefficients of the proposed network in ET, WT, and TC are increased by 1.75% and 1.55%, respectively, and decreased by 0.56% in WT, but our Hausdorff95 distances are significantly reduced in ET and TC, and its parameter amount is 7.76 times that of MVKS-Net. Even with significant compression of the parameters and calculations, MVKS-Net can still have similar or better segmentation performance. Compared with the advanced HDC-Net, although the number of parameters and calculations of MVKS-Net has increased, the Dice coefficients on ET, WT, and TC increase by 1.74%, 0.5%, and 2.19%, respectively, and the Hausdorff95 distances are reduced by 11.24 mm, 1.99 mm, and 2.98 mm on ET, WT, and TC, respectively. Therefore, MVKS-Net significantly improves segmentation accuracy in terms of maintaining memory usage and training speed at levels equal equivalent to HDC-Net, which indicates that our proposed network has more powerful feature learning capabilities.

It can be seen from Table 5 that compared with the segmentation effect of the classic 3D U-Net, the Dice coefficients of MVKS-Net in ET, WT, and TC are increased by 9.4%, 5.41%, and 3.99%, and the Hausdorff95 distances are reduced by 26.4 mm, 5.75 mm, and 3.57 mm, respectively. However, 3D U-Net has 32.42× more parameters and 1640.97× more computations. In addition, our network has better performance than V-Net, Residual U-Net, and Attention U-Net. Compared with the advanced SwinBTS, MVKS-Net has improved the Dice coefficients of 0.8%, 0.46%, and 2.75% in ET, WT, and TC, respectively. Compared with the ME-Net, MVKS-Net has improved the Dice coefficients of 8.16%, 1.52%, and 9.05% in ET, WT, and TC, respectively. Compared to the AEMA-Net, our network has a 1.16% improvement in the Dice coefficients in ET, WT is basically the same, and TC is reduced by 0.85%. However, our Hausdorff95 distances are reduced by 7.82 mm, 0.08 mm, and 1.66 mm, respectively. In contrast, our network is lighter. We also compared the brain tumor segmentation results from multiple methods, such as the non-lightweight CASPIANET++, which showed competitive results in both the Dice coefficient and Hausdorff95 distances.

Table 6 shows the experimental results of different networks on the BraTS2018 validation dataset. The brain tumor segmentation results of 3D U-Net, 3D-ESPNet, DMF-Net, and HDC-Net were obtained by retraining the networks. As can be seen from Table 5, MVKS-Net has higher Dice coefficients than 3D-UNet and 3D-ESPNet. Compared with DMF-Net, MVKS-Net has a 0.11% lower Dice coefficient in TC, but DMF-Net has 6.72× more parameters than ours. Compared with HDC-Net, our network has apparent advantages in the segmentation accuracy of WT, in which the Dice coefficient is increased by 0.81%. Compared with the latest model proposed by Akbar et al., the Dice coefficients of MVKS-Net were improved by 2.17%, 0.41%, and 3.62% for ET, WT, and TC, respectively, and the Hausdorff95 distances were reduced by 1.59 mm, 5.18 mm, and 1.04 mm, respectively. The experimental results further validate that MVKS-Net shows competitive results in terms of both Dice coefficients and Hausdorff95 distances.

**Figure 10:**
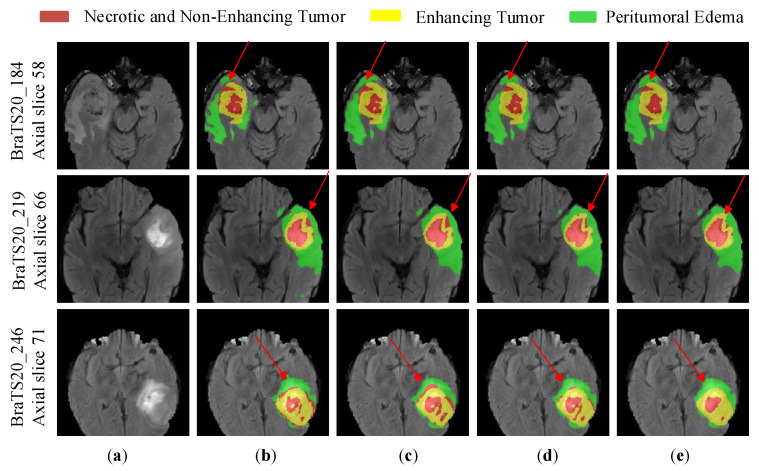
Segmentation results of brain tumor images by different networks. (**a**) is the modality of Flair, (**b**) stands for ground truth, (**c**) stands for MVKS-Net, (**d**) stands for HDC-Net, (**e**) stands for DMF-Net. The yellow area is the enhancing tumor area. The red area is the necrotic and non-enhancing area. The green area is the peritumoral edema area.

In order to visualize the performance of MVKS-Net, we selected the most competitive lightweight networks, DMF-Net and HDC-Net, for visualizing the segmentation results. Figure 10 shows the three random cases in the BraTS 2020 training set from top to bottom. As shown in Figure 10d, HDC-Net can segment the general tumor shape, but sporadic lesion areas are still not finely segmented. The segmentation results of DMF-Net are shown in Figure 10e. Large false-negative regions appear in the segmentation results, i.e., there are large lesion regions that are not detected, and the segmentation results in the small target tumor regions have a large gap with the true values. The segmentation results of MVKS-Net are shown in Figure 10c. Compared with other models, our network segmented the tumor region with the highest similarity to the true-value labels, especially for the necrotic area, indicating that our network improved the segmentation quality of brain tumor subregions.

In summary, MVKS-Net segmentation accuracy is more competitive, and the overall network has fewer parameters and lower computational power consumption, so MVKS-Net is an efficient brain tumor segmentation network.

## 5. Conclusions

This study proposes an efficient multimodal brain tumor segmentation network called MVKS-Net. By using multi-view fusion convolution and kernel-sharing dilated convolution instead of standard convolution, the average Dice coefficients of ET, WT, and TC on the BraTS2020 validation set can reach 78.16%, 89.52%, and 83.05%, respectively, with only 0.5 M parameters and 28.56 G floating-point operations. The results show that our network also has high segmentation accuracy and low arithmetic resource consumption, which can provide a strong reference for clinicians to perform brain tumor segmentation.

The proposed network in this paper deeply exploits the characteristics of brain tumor images. By using hierarchical multi-view fusion convolution with an ensemble discrimination idea, the segmentation accuracy of brain tumors can be further improved. In addition, kernel-sharing dilated convolution combines the scale adaptive idea of tumor feature similarity, which can combine different scale features to adapt to complex tumor boundaries. MVKS-Net is grounded in MR image information, and we can further consider the positional correlation between the three regions of the brain tumor with the inclusion relationship. The positional correlation between the edematous regions of brain tumors, tumor cores, and enhanced tumors can be further explored to introduce into the model. In addition, the model’s accuracy needs to be improved due to the limitation of the small size of the current dataset. In future work, we can consider extending the lightweight, efficient, and concise MVKS-Net to weakly supervised scenarios.

## 6. Future Work

Although the network proposed in this paper has achieved certain results and enhancements, there are still aspects that can be further refined and improved. For better follow-up, future research could be carried out in the following directions:

Firstly, due to the limitation of computing power resources, the input of our network uses cropped image blocks of MRI brain tumor images, which makes the feature information of tumor images learned by the network incomplete. In the future, larger-sized input image blocks can be used to obtain more comprehensive tumor information and improve network segmentation accuracy.

Secondly, our network is a direct concatenation of the four modalities of brain tumor MR images. However, each modality reflects different tissue information of brain tumors to different degrees, and taking full advantage of the complex relationships between brain tumor modalities will help guide the model for segmentation. In the future, multimodal fusion strategies can be considered to learn complex nonlinear complementary information between modalities in order to efficiently fuse and refine multimodal features.

## Figures and Tables

**Table 1:** The network structure of MVKS-Net, where Conv 3 × 3 × 3 (1 × 1 × 1, 1 × 3 × 3) Block stands for 3 × 3 × 3 (1 × 1 × 1, 1 × 3 × 3) convolution, Synchronized Batch Normalization, ReLU. ConvTranspose 3 × 3 × 3 Block stands for deconvolution with kernel size 3 × 3 × 3.

	Name	Details	Input	Output
	HDC_transform	Down_sampling	4 × 128 × 128 × 128	32 × 64 × 64 × 64
	Conv 3 × 3 × 3	Conv 3 × 3 × 3 Block	32 × 64 × 64 × 64	32 × 64 × 64 × 64
	ACSF_module1	Conv 1 × 1 × 1 Block, ACSF Conv × 3, Conv 1 × 1 × 1 Block, Conv 1 × 3 × 3 Block	32 × 64 × 64 × 64	32 × 64 × 64 × 64 (x1)
Encoding	Conv_down1	Conv 3 × 3 × 3 Block (stride = 2)	32 × 64 × 64 × 64	32 × 32 × 32 × 32
	ACSF_module2	Conv 1 × 1 × 1 Block, ACSF Conv × 3, Conv 1 × 1 × 1 Block, Conv 1 × 3 × 3 Block	32 × 32 × 32 × 32	32 × 32 × 32 × 32 (x2)
	Conv_down2	Conv 3 × 3 × 3 Block (stride = 2)	32 × 32 × 32 × 32	32 × 16 × 16 × 16
	ACSF_module3	Conv 1 × 1 × 1 Block, ACSF Conv × 3, Conv 1 × 1 × 1 Block, Conv 1 × 3 × 3 Block	32 × 16 × 16 × 16	32 × 16 × 16 × 16 (x3)
	Conv_down3	Conv 3 × 3 × 3 Block (stride = 2)	32 × 16 × 16 × 16	32 × 8 × 8 × 8
	KSDC_module	KSDC_module	32 × 8 × 8 × 8	32 × 8 × 8 × 8
	Conv_up1	ConvTranspose 3 × 3 × 3 Block (stride = 2)	32 × 8 × 8 × 8	32 × 16 × 16 × 16 (y1)
	Skip_connection1	torch.cat (y1, x3)	32 × 16 × 16 × 16	64 × 16 × 16 × 16
	ACSF_module4	Conv 1 × 1 × 1 Block, ACSF Conv × 3, Conv 1 × 1 × 1 Block, Conv 1 × 3 × 3 Block	64 × 16 × 16 × 16	32 × 16 × 16 × 16
	Conv_up2	ConvTranspose 3 × 3 × 3 Block (stride = 2)	32 × 16 × 16 × 16	32 × 32 × 32 × 32 (y2)
Decoding	Skip_connection2	torch.cat (y2, x2)	32 × 32 × 32 × 32	64 × 32 × 32 × 32
	ACSF_module5	Conv 1 × 1 × 1 Block, ACSF Conv × 3, Conv 1 × 1 × 1 Block, Conv 1 × 3 × 3 Block	64 × 32 × 32 × 32	32 × 32 × 32 × 32
	Conv_up3	ConvTranspose 3 × 3 × 3 Block (stride = 2)	32 × 32 × 32 × 32	32 × 64 × 64 × 64 (y3)
	Skip_connection1	torch.cat (y3, x1)	32 × 64 × 64 × 64	64 × 64 × 64 × 64
	ACSF_module6	Conv 1 × 1 × 1 Block, ACSF Conv × 3, Conv 1 × 1 × 1 Block, Conv 1 × 3 × 3 Block	64 × 64 × 64 × 64	32 × 64 × 64 × 64
	Upsample4	Up_sampling	32 × 64 × 64 × 64	32 × 128 × 128 × 128
	Conv_output	Conv 1 × 1 × 1	32 × 128 × 128 × 128	4 × 128 × 128 × 128
	Softmax	Softmax	4 × 128 × 128 × 128	4 × 128 × 128 × 128

**Table 2:** Parameter settings during model training.

Parameter	Value
Weight decay	$10^{-5}$
Initial learning rate	$10^{-3}$
Optimizer	Adam
Epoch	900
Batch size	8

**Table 3:** Ablation study of the method on the BraTS 2020 validation set.

Model	Params (M)	FLOPs (G)	Dice Coefficient (%)			Hausdorff95 (mm)		
			ET	WT	TC	ET	WT	TC
HDC	0.29	25.62	76.42	89.02	80.86	35.82	9.61	13.02
HDC + ACSF	0.32	28.08	77.73	89.46	82.20	30.21	6.21	12.63
HDC + KSDC	0.47	26.11	77.53	89.11	81.34	27.58	7.24	13.53
HDC + ACSF + KSDC	0.50	28.56	78.16	89.52	83.05	24.58	7.62	10.04

**Table 4:** Comparison of segmentation results of various lightweight networks on BraTS2020 validation set.

Model	Params (M)	FLOPs (G)	Dice Coefficient (%)			Hausdorff95 (mm)		
			ET	WT	TC	ET	WT	TC
3D-ESPNet [29]	3.36	76.51	69.0	87.10	78.60	31.29	**7.10**	14.61
DMF-Net [32]	3.88	27.04	76.41	**90.08**	81.50	35.17	7.17	12.17
HDC-Net [37]	**0.29**	**25.62**	76.42	89.02	80.86	35.82	9.61	13.02
MVKS-Net (Ours)	0.50	28.56	**78.16**	89.52	**83.05**	**24.58**	7.62	**10.04**

**Table 5:** Comparison of segmentation results of various non-lightweight networks on the BraTS2020 validation set. A (-) denotes that the results are not reported.

Model	Params (M)	FLOPs (G)	Dice Coefficient (%)			Hausdorff95 (mm)		
			ET	WT	TC	ET	WT	TC
3D U-Net [38]	16.21	1669.53	68.76	84.11	79.06	50.98	13.37	13.61
V-Net [36]	-	-	68.97	86.11	77.90	43.52	14.49	16.15
Residual U-Net [39]	-	-	71.63	83.46	76.47	37.42	12.34	13.11
Attention U-Net [40]	-	-	71.83	85.57	75.96	32.94	11.91	19.43
SwinBTS [41]	-	-	77.36	89.06	80.30	26.84	8.56	15.78
ME-Net [42]	-	-	70.0	88.0	74.0	38.6	6.95	30.18
Akbar et al [43]	-	-	72.91	88.57	80.19	31.97	10.26	13.58
CASPIANET++ [44]	-	-	77.37	89.26	81.56	27.13	7.22	9.45
NoNew-Net [45]	12.42	296.82	76.8	89.1	81.9	38.35	**6.32**	**7.34**
MVKS-Net (Ours)	**0.50**	**28.56**	**78.16**	**89.52**	**83.05**	**24.58**	7.62	10.04

**Table 6:** Comparison of segmentation effects of various networks on the BraTS2018 validation set. A (-) denotes that the results are not reported.

Model	Params (M)	FLOPs (G)	Dice Coefficient (%)			Hausdorff95 (mm)		
			ET	WT	TC	ET	WT	TC
3D U-Net [38]	16.21	1669.53	75.96	88.53	71.77	6.04	17.10	11.62
3D-ESPNet [29]	3.36	76.51	73.70	88.30	81.40	5.30	5.46	7.85
DMF-Net [32]	3.88	27.04	78.1	89.9	**83.5**	3.38	4.86	7.74
HDC-Net [37]	**0.29**	**25.62**	79.13	89.19	83.03	**2.27**	5.77	**7.45**
Akbar et al. [43]	-	-	77.71	89.59	79.77	3.90	9.13	8.67
Zhang et al. [46]	-	-	78.2	89.6	82.4	3.57	5.73	9.27
MVKS-Net (Ours)	0.50	28.56	**79.88**	**90.00**	83.39	2.31	**3.95**	7.63

## Data Availability

Data will be made available on request from the corresponding author.
